# Extraction Efficiency of Shosaikoto (Xiaochaihu Tang) and Investigation of the Major Constituents in the Residual Crude Drugs

**DOI:** 10.1155/2012/890524

**Published:** 2012-09-02

**Authors:** Megumi Sumino, Yuko Saito, Fumio Ikegami, Yoshiro Hirasaki, Takao Namiki

**Affiliations:** ^1^Center for Environment, Health and Field Sciences, Chiba University, 6-2-1 Kashiwanoha, Kashiwa 277-0882, Japan; ^2^Department of Japanese Oriental (Kampo) Medicine, Graduate School of Medicine, Chiba University, 1-8-1 Inohana, Chuo-ku, Chiba 260-8670, Japan

## Abstract

Shosaikoto (Xiaochaihu Tang) is one of the frequently used traditional herbal medicines (Kampo medicines, Japan). To realize the effective use of precious crude drugs, we investigated the major constituents in the residual crude drugs after decoction and aimed for extraction efficiency of shosaikoto with regard to the extract, tannin content, and major constituents. We found that the residual crude drugs had large amounts of compounds, especially saikosaponin b_2_, which had a 78.3% yield compared to that in the first decoction. The extraction efficiency increased when decoction time and volume of water increased. Both increases had an additive effect on the yield of the extract and saikosaponin b_2_ in particular. We also found that the size of crude drug pieces that are available in Japanese markets is suitable for decoction because of quick permeation of water. From our study, the second decoction may be a valuable contribution to medical treatment and of effective use of crude drugs. Moreover, time and volume of water should be increased when patients have trouble in preparing a decoction. Our study revealing the factors that influence the extraction efficiency of shosaikoto will be the basis for empirical evidence about decocting Kampo medicine.

## 1. Introduction

Kampo medicine (Japanese traditional medicine) originally developed in Japan under the influence of traditional Chinese medicine in the 6th and 7th centuries. In recent times, Kampo medicines have been used by more than 80% of Japanese doctors, since many Kampo products came to be included under the coverage of the public health insurance system in 1976 [1, 2]. Kampo treatment aims at normalizing the distortion of mind and body in patients and improving their quality of life (QOL) rather than curing the local disease. Thus, in parallel to the increase of chronic, lifestyle-related, and complex diseases such as high blood pressure, lipid abnormality symptoms, and diabetes, patients have increasingly turned to traditional Kampo treatment for relief of their symptoms. 

Kampo formulae contain several crude drugs that have been screened through many years of use. At present, 172 crude drugs are listed on the Japanese Pharmacopoeia, 16th edition. We depend on importing more than 80% of the resource from China, but nature resources in China are limited and agriculture has been gradually declining in recent years [3]. Moreover, Chinese domestic demand for crude drugs is growing, and personnel expenses are mounting, which affects the price of the crude drugs. This situation will affect the continuous acquisition of the natural resources that are necessary in Japan. To increase the domestic production of original plant sources of crude drugs, technical studies to determine how to produce high-quality drugs are in progress. For example, a fine strain of Glycyrrhiza was cultivated by a hydroponic production system and in plastic tubes [4–6].

It is also important to extract crude drugs of Kampo medicines efficiently. The decoction method is described in classic texts such as the Shokanron (Shanghanlun in Chinese), a text of instructions on the diagnosis and treatment of acute febrile disease called “shanghan,” that is thought to have been completed during the Han dynasty (206 BC–220 AD). It is based on experience but not well-grounded scientifically, and there have been few studies on the quality of decoction.

Shosaikoto (Xiaochaihu Tang) is popular and clinically used for the treatment of various feverish diseases such as the common cold, pneumonia, and hepatic function diseases, and it has been studied pharmacologically [7–10]. Shosaikoto contains precious and expensive crude drugs such as Bupleurum Root, Scutellaria Root, and Ginseng. The influence of the quality of water on the decoction of shosaikoto has been reported [11, 12]. In this study, we investigated the quantitative analysis of major constituents, yield and tannin content in the residual crude drugs of shosaikoto. The aim of the present study was to investigate the effect of several conditions such as decoction time, volume of water and size of crude drug pieces on extraction efficiency to contribute to saving natural resources.

## 2. Materials and Methods

### 2.1. Plant Materials

 Shosaikoto (daily dose) is composed of seven crude drugs: Bupleurum Root (*Bupleurum falcatum* L., 7.0 g), Pinellia Tuber (*Pinellia ternata *Breit., 5.0 g), Scutellaria Root (*Scutellaria baicalensis* Georgi, 3.0 g), Jujube Fruit (*Zizyphus jujuba* Miller var.* inermis* Rehder, 3.0 g), Ginseng (*Panax ginseng* C.A. Meyer, 3.0 g), Glycyrrhiza (*Glycyrrhiza uralensis* Fisher, 2.0 g), and Ginger (*Zingiber officinale* Roscoe, 1.0 g) [13]. All crude drugs used in this study met the criteria of the Japanese Pharmacopoeia, 16th edition. 

Lot A: Bupleurum Root (Sichuan, China, Lot. 004211005, 2 to 3 mm pieces), Pinellia Tuber (Sichuan, China, Lot. 009110012, 5 mm pieces), Scutellaria Root (mixed with wild plant in Shanxi and cultivated plant in Shaanxi, China, Lot. 001111002, 2 to 3 mm pieces), Jujube Fruit (Hebei, China, Lot. 007111006, 5 mm pieces), and Glycyrrhiza (Neimenggu, Lot. 002010018, 5 mm pieces) were purchased from Tochimoto Tenkaido (Osaka, Japan). Ginseng (Jilin, China, Lot. OA50403, 5 mm pieces) and Ginger (Yunnan, China, Lot. OBK0227, 2 to 3 mm pieces) were from Uchida Wakanyaku (Tokyo, Japan). 

Lot B: Bupleurum Root (Sichuan, China, Lot. 1003C004203, cut 30–40 mm long), Pinellia Tuber (Sichuan, China, Lot. 0907C009101, crushed 8 mm pieces), Scutellaria Root (cultivated plant in Shaanxi, China, Lot. 0907C001102, sliced 2 mm thick), Jujube Fruit (Hebei, China, Lot. 007111017, torn and seeds removed), and Glycyrrhiza (Neimenggu, Lot. 1009C002001, sliced 4 mm thick) were purchased from Tochimoto Tenkaido (Osaka, Japan). Ginseng (Jilin, China, Lot. TA362125, sliced 2 mm thick) and Ginger (Yunnan, China, Lot. UA322606, sliced 2 mm thick) were from Uchida Wakanyaku (Tokyo, Japan). Crude drugs of Lot B were used for preparing large crude drug pieces as described in the Chinese Pharmacopoeia, 9th edition (2010), and matching products in the Chinese market. The Chinese Pharmacopoeia instructs users to slice Scutellaria Root and Ginseng at a thickness of 1-2 mm, to slice Bupleurum Root, Ginger, and Glycyrrhiza at a thickness of 2–4 mm to crush Pinellia Tuber and to tear Jujube Fruits when any of these are designated as crude drug pieces.

### 2.2. Reagents

The authentic specimens of saikosaponin b_2_, saikosaponin d, glycyrrhizin, and baicalin were purchased from Wako Pure Chemical Industries (Osaka, Japan). Phosphoric acid, acetic acid, sodium dihydrogenphosphate dehydrate and potassium sodium (+)-tartrate tetrahydrate from Kanto Chemical Co., Inc. (Tokyo, Japan), iron (II) sulfate heptahydrate from Wako Pure Chemicals Industries (Osaka, Japan), and ethyl 3,4,5-trihydroxybenzoate from Alfa Aesar (Wako) were used. Acetonitrile of high-performance liquid chromatography (HPLC) grade was purchased from Sigma-Aldrich (Tokyo, Japan). Methanol of HPLC grade was purchased from Wako.

Distilled water was prepared by the purification system of Millipore. Tap water was used for preparing decoctions (pH 7.2, Ca^2+^ : 24.7 mg/L, Mg^2+^ : 6.3 mg/L, measured using a polarized Zeeman atomic absorption spectrophotometer, Z-5300, HITACHI).

### 2.3. Preparation of Decoction

A daily dose of shosaikoto and 400 mL of tap water were put in a heat-resistant glass pot with a lid. The pot was heated on a heater (350W, EK-SA10, Tochimoto Tenkaido, Osaka, Japan), and it took around 12 minutes for the mixture to boil. The first decoction was prepared by continuously boiling for 30 min followed by paper filtration. The residue was decocted again by adding 300 mL of tap water and boiling for 30 min followed by paper filtration. The filtrate was immediately used for the tannin quantitative assay. Each portion of the filtrate was freeze-dried to weigh the extract (Figure 2). Powdered drugs were decocted in a paper bag (22.0 g/m^2^, 0.068 mm, Miki Tokushu Paper Mfg. Co., Ltd., Japan) due to the difficulty in the following filtration and the poorly-reproducible results when decocting them directly.

### 2.4. Tannin Quantitative Assay

The tannin content in a decoction was analyzed by the colorimetric method using chelating properties with polyphenols and ferrous tartrate [14]. This quantitative method is widely and officially used for Japanese green tea because of its good reproduction and stability of the created chelates. Briefly, ferrous tartrate reagent was prepared containing 0.1% (w/v) iron (II) sulfate heptahydrate and 0.5% (w/v) potassium sodium (+)-tartrate tetrahydrate in water. Equal amounts of decoction and ferrous tartrate reagent and 3 times volume of 0.05 M phosphate buffer (pH 7.5) were mixed. The absorbance of the created chelates was measured at 540 nm by spectrophotometer (U-2000, HITACHI with auto sampler AS-3000, HITACHI). The value was expressed as the equal amount of ethyl 3,4,5-trihydroxybenzoate, the positive compound.

### 2.5. HPLC Analysis

HPLC analysis was conducted using the following instruments: Column: TSK gel ODS-80Ts (4.6 i.d. × 150 mm, TOSOH) for saikosaponin b_2_, saikosaponin d and baicalin, Inertsil ODS-3 (4.6 i.d. × 150 mm, GL Sciences Inc.) with a guard column (Inertsil, 4.6 i.d. × 50 mm, GL Sciences Inc.) for glycyrrhizin. Column oven: L-5025 (HITACHI), Pump: L-2130 (HITACHI), UV detector: L-4000 (HITACHI), Integrator: D-2500 Chromato-Integrator (HITACHI).

### 2.6. HPLC Conditions

The HPLC conditions were determined based on the method for testing shosaikoto given in the Japanese Pharmacopoeia, 16th edition. Saikosaponin b_2_, baicalin, and glycyrrhizin are listed as principal compounds for quality control as follows: Saikosaponin b_2_ (mobile phase, flow rate, wave length): 0.05 mol/L NaH_2_PO_4_/CH_3_CN = 5/3, 1 mL/min, 254 nm. Baicalin: 0.5% H_3_PO_4_/CH_3_CN = 19/6, 1 mL/min, 277 nm. Glycyrrhizin: 2% acetic acid/CH_3_CN = 13/7, 1 mL/min, 254 nm. Saikosaponin d: CH_3_CN/H_2_O = 2/3, 1 mL/min, 206 nm. The column temperature was 40°C in all experiments.

### 2.7. Statistical Analysis

Each value represents the mean ± standard deviation (SD), *n* = 4–6. Differences were considered to be statistically significant when the *P* value was less than 0.05 by the Tukey test or Student's *t*-test.

## 3. Results

### 3.1. Comparison of the Properties of First and Second Decoctions

The volume of each decoction and the yield of the freeze-dried extract are shown in Table 1. The first decoction was prepared in the range of 200 to 300 mL, which can be reasonably divided into two or three intakes a day. The second decoction was prepared with a smaller volume of water because the residual crude drugs absorbed the first decoction and the extract was thought to be thinner than the first one. The freeze-dried extract of the second decoction was 25.7% of that of the first decoction. Tannin content in the second decoction was 35.3% of that of the first decoction when expressed as ethyl gallate equivalent.

**Figure 1:**
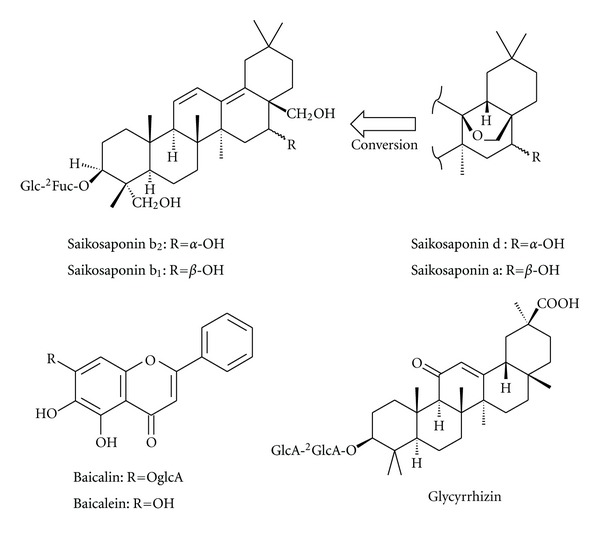
Structure of principal compounds in shosaikoto.

**Figure 2:**
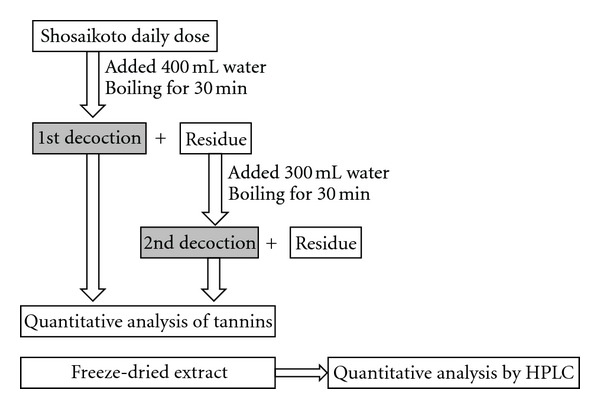
Preparation of first and second decoctions.

 Regarding principal compounds (structures are shown in Figure 1), saikosaponin b_2_, glycyrrhizin, and baicalin as well as saikosaponin d were analyzed in first and second decoctions by HPLC, and the contents are shown in Table 1. Saikosaponin b_2_, glycyrrhizin, and baicalin are designated as the principle compounds for quality control for shosaikoto in the Japanese Pharmacopoeia, 16th edition. Saikosaponin d was analyzed in this study because saikosaponin b_2_ is generated from it during decoction. The yields of compounds in the second decoction were high compared with those of the extract, especially saikosaponin b_2_ (78.3%), saikosaponin d (58.8%) and glycyrrhizin (49.2%). The ratio of tannin in the second decoction was increased to some extent. The yield of baicalin was 31.0%, which is close to the yield of extract, 25.7%.

### 3.2. Influence of Time and Volume of Water on the Extraction Efficiency

To have to decoct two times can be troublesome and time consuming, so we tried to determine the optimum conditions by decocting shosaikoto. The decoction was prepared with 400 mL of water and boiling for 30 minutes according to the usual method. To understand the influence of time and volume of water, we then increased the boiling time and/or the volume of water. The results are shown in Table 2. The extract gained using the usual method, 6.31 ± 0.11 g, increased significantly to 7.16 ± 0.13 g when the volume of water was increased to 600 mL, and to 7.50 ± 0.13 g, 1.2 times as heavy as the control, when the time and water were increased to 50 min and 600 mL, respectively. Tannin, glycyrrhizin and baicalin showed the same tendency as the extract, and the increase of both time and water had an additive effect on the extraction efficiency. Saikosaponin d is converted to saikosaponin b_2_ via hydrolysis and dehydration during the decoction. In this case, time prolongation and the increase of both time and water significantly stimulated the production of saikosaponin b_2_ (e.g., 3.73 ± 0.32 mg, 5.17 ± 0.45 mg). An increase of water did not have any effect on the amount of saikosaponin b_2_.

### 3.3. Influence of Size of Crude Drug Pieces on the Extraction Efficiency

To study the effect of the different size of pieces of crude drugs on the extraction efficiency, we prepared crude drug pieces in three different sizes, commercial (2 to 5 mm pieces), powdered, and large. Usually, crude drugs in Japan are subjected to coarse cutting (4.75 mm sieve) for leaves, flowers, and herbs, medium cutting (2.8 mm) for wood, bark, roots, and rhizomes, and fine cutting (2.0 mm) for seeds and fruits, as described in the Japanese Pharmacopoeia, 16th edition, though the actual size is sometimes different depending on the property of the crude drug and what is required for stability. In contrast, crude drug pieces in China are generally larger than those in Japan, and most of them are sliced rather than cut into pieces. The size is regulated by the Chinese Pharmacopoeia, 9th edition. In the present study, large crude drug pieces were newly prepared using lot B, referring to the products from the Chinese markets and the Chinese Pharmacopoeia. Powdered crude drugs were prepared using a blender and immediately used for boiling. The decoction of powdered drugs became widespread beginning in the Sung dynasty, because less of the drug was needed than for the conventional decoction and thus the precious drugs could be saved and ordinary people could receive their benefit [15].

**Figure 3:**
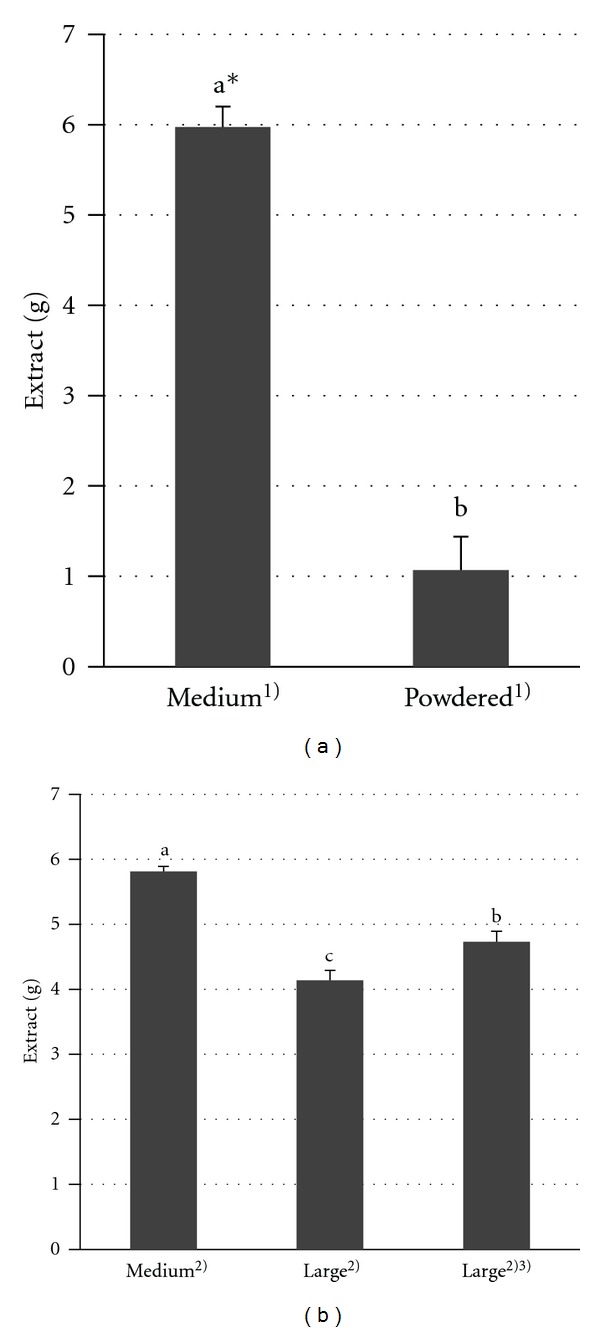
Influence of the size of crude drugs on the extraction efficiency. *The results of Student's *t*-test or the Tukey test are indicated by different letters where values differ significantly at *P* < 0.05 (*n* = 4). (1) Lot A, decocted in a paper bag. (2) Lot B. (3) Soaked in water (30 min).

A comparison of the extracts is shown in Figure 3. Decocting powdered drugs directly made the following filtration very difficult, and the data were not reproducible (data not shown), so powdered drugs were decocted in a paper bag and compared to the results for crude drugs in a paper bag. The bags were made of thin Japanese paper that passes water easily but does not pass powdered drugs. The extract with a paper bag was as heavy as that without a paper bag (5.97 ± 0.23 g, *n* = 4). The extract of powdered drugs (1.07 ± 0.37 g) was significantly low and 17.9% of that of the control (lot A, 5.97 ± 0.23 g). The extract of large crude drug pieces (4.14 ± 0.15 g) was also low and 71.8% of that of the control (lot B, 5.81 ± 0.08 g). After soaking large crude drug pieces in water for 30 min, we found that the extract (4.73 ± 0.16 g, 81.4% of the control, lot B) was significantly increased compared to the extract from pieces that were not soaked.

## 4. Discussion

To conserve the natural resources, especially original plant sources of crude drugs, it is vital to extract crude drugs of Kampo medicines efficiently. First, we studied the remaining principal compounds in the residual crude drugs of shosaikoto: saikosaponin b_2_, glycyrrhizin and baicalin, as well as saikosaponin d, by decocting twice. We found that the residue contains considerable compounds. The yields of saikosaponin b_2_, saikosaponin d, and glycyrrhizin were especially high in the second decoction. The authors of an earlier study noted that Bupleurum root had low extraction efficiency in view of saikosaponins when using water [16]. Thus, redecoction may gain the medicinal components of Bupleurum root as well as other crude drugs, and total intake of decoctions may have a medicinal effect. In China, decoction is often done two or three times, with the extracts combined [17]. One of our goals is to carefully study when decoctions are prepared containing crude drugs with a lot of volatile oils such as Mentha Herb, Perilla Herb, and Cinnamon Bark, because volatile oils can easily evaporate during long decoctions.

In the present study, we investigated the effect of decocting time, volume of water and the size of crude drug pieces on the extraction efficiency of shosaikoto. The authors of an earlier study indicated the effect of the property of water on the extract of shosaikoto, saying that differences in the hardness and pH of water may affect the extraction, which may further change the effectiveness of a drug [11]. They also mentioned that a weak acidic condition promoted the saikosaponin b_2_ production, while a neutral condition with the addition of Ostreae Testa and Fossilia Ossis Mastodi suppressed the production, and mineral water may act like Ostreae Testa and Fossilia Ossis Mastodi, which contain a lot of calcium salts. On the other hand, the authors of a more recent study said that the hardness of water had minimal influence on the extraction of shosaikoto [12].

In the present study, the increase in time and volume of water influenced the extract and constituents. Time prolongation significantly increased the amount of saikosaponin b_2_. A large amount of water increased the extract. We found that each compound was influenced by different factors. An additive effect was observed on every value when both time and volume of water were increased. In particular, the extract and saikosaponin b_2_ was 1.2 and 1.9 times as heavy as the control, respectively. Thus, we conclude that the time and volume of water should be increased to improve extraction efficiency. When patients decoct with less water or finish decocting earlier than the designated time, the instructions, based on this study, should tell them the potential medicinal effect and thus be useful for understanding the importance of decoction method. Our findings will be important and useful background information for patients and doctors as well as pharmacists.

To clarify the effect of the different size of crude drug pieces on the extraction efficiency, we compared extraction using three piece sizes. Large crude drug pieces had to be newly prepared, so the control was prepared again using the crude drugs from lot B. Large crude drug pieces, commercial-sized crude drug pieces and powdered drugs were decocted under the same conditions, respectively. The extract of powdered drugs was very low, and this fact was contrary to our expectations because the surface area must increase by powdering, which should have resulted in extraction efficiency. The residue was muddy and brown, though the residue of general crude drugs was decolorized. Thus, we suspected that constituents remained in the residue of powdered drugs and that it might be a good idea to take the muddy part as well, though the material of the paper in view of the extraction efficiency and the extent of bioavailability should be examined further.

 The extract of large crude drug pieces was significantly lower than that of the control. To exclude the possibility that the pieces were too large to absorb water into the inner material, we soaked the pieces in water for 30 min and checked to see if they were wet on the inside. The extract was significantly increased compared to that without soaking. Thus, it is possible that water permeation is important to increase the extraction efficiency.

## 5. Conclusions

 In this study, we revealed the residual constituents from crude drugs and the factors that influence the extraction efficiency of shosaikoto. The residual crude drugs contained considerable compounds that, by taking a second decoction, may have a medicinal effect like that of the crude drugs. In the case of medical instruction to patients, time and volume of water should be increased when patients have trouble in preparing decoctions. We also found that the size of crude drugs that are available in Japanese markets is suitable for decoction because of quick permeation of water. This is a fundamental study concerning a decoction method, but we believe this information will play an instrumental part in establishing scientific grounds for empirical evidence of Kampo medicine. Further study will be needed on other Kampo medicines to accumulate factors that influence the extraction efficiency.

## Figures and Tables

**Table 1:** Comparison of first and second decoctions in extract, tannin, and four principal compounds.

	1st decoction	2nd decoction	(2nd/1st decoction) %
Decoction (mL)	276.6 ± 4.4	229.6 ± 3.4	—
Extract (g)	6.31 ± 0.11	1.62 ± 0.19	25.7
Tannin (mg)*	112.8 ± 11.5	39.8 ± 4.0	35.3
Saikosaponin b_2_(mg)	2.67 ± 0.36	2.09 ± 0.11	78.3
Glycyrrhizin (mg)	31.04 ± 4.83	15.27 ± 3.35	49.2
Baicalin (mg)	296.1 ± 13.8	91.8 ± 13.4	31.0
Saikosaponin d (mg)	2.89 ± 0.78	1.70 ± 0.21	58.8

**Table 2:** Influence of various conditions on the extraction efficiency.

Condition	Water volume	Decoction time	Number of experiment	Total extract (g)	Tannin (mg, ethyl gallate equivalent)	Saikosaponin b_2_ (mg)	Glycyrrhizin (mg)	Baicalin (mg)	Saikosaponin d (mg)
Control	400 mL	30 min	6, 5 (tannin)	6.31 ± 0.11c^*∗*^	112.8 ± 11.5bc	2.67 ± 0.36c	31.1 ± 4.3b	296.1 ± 13.8b	2.89 ± 0.78a
Water increase	600 mL	30 min	4	7.16 ± 0.13b	126.8 ± 9.2ab	2.58 ± 0.15c	35.0 ± 1.2ab	325.5 ± 28.5ab	3.49 ± 0.66a
Time prolongation	400 mL	50 min	4	6.44 ± 0.09c	103.9 ± 4.5c	3.73 ± 0.32b	32.9 ± 1.5ab	303.7 ± 30.3b	3.20 ± 0.31a
Time and water increase	600 mL	50 min	4	7.50 ± 0.13a	132.5 ± 4.0a	5.17 ± 0.45a	37.9 ± 3.1a	357.1 ± 15.7a	3.76 ± 0.39a

Each value represents the mean ± SD.

*The results of the Tukey test (within columns) are indicated by different letters where values differ significantly at *P* < 0.05.

## References

[1] (2011). TSUMURA cooperate Social Responsibility Report 2011.

[2] Akiba T (2010). History of Kampo extracts for medical use. *Kampo Medicine*.

[3] Kang D (2011). Resources of medicinal herbs. Considerations for the necessity of domestic production. *Kampo & the Newest Therapy*.

[4] Ozaki K, Shibano M, Kusano G, Watanabe H (2007). Aim for production of Glycyrrhizae Radix in Japan (1). A novel cultivation method of Glycyrrhiza uralensis Fisher. *Shoyakugaku Zasshi*.

[5] Ozaki K, Shibano M, Kusano G, Watanabe H (2007). Aim for production of Glycyrrhizae Radix in Japan (2). Selection of pharmaceutically fine strains from Glycyrrhiza uralensis Fisher. *Shoyakugaku Zasshi*.

[6] Jiho editorial department (2011). National Institute of Biomedical Innovation, Kajima Corporation and Chiba University developed the system for the stable domestic production of high-quality Glycyrrhiza uralensis (glabra). *Pharm Tech Japan*.

[7] Kaji M, Kashiwagi S, Yamakido M (2001). A double-blind, placebo-controlled study of TSUMURA Shosaikoto (TJ-9) for common cold. *Rinsho to Kenkyu*.

[8] Ohtake N, Suzuki R, Daikuhara H (2000). Modulation of lung local immune responses by oral administration of a herbal medicine Sho-saiko-to. *International Journal of Immunopharmacology*.

[9] Taira Z, Yabe K, Hamaguchi Y (2004). Effects of Sho-saiko-to extract and its components, Baicalin, baicalein, glycyrrhizin and glycyrrhetic acid, on pharmacokinetic behavior of salicylamide in carbon tetrachloride intoxicated rats. *Food and Chemical Toxicology*.

[10] Ikegami F, Sumino M, Fujii Y, Akiba T, Satoh T (2006). Pharmacology and toxicology of Bupleurum root-containing Kampo medicines in clinical use. *Human and Experimental Toxicology*.

[11] Sakata K, Kim SJ, Yamada H (2000). Effects of commercially available mineral waters on decoction of Kampo Medicines. *Kampo Medicine*.

[12] Honma S, Ogawa A, Kobayashi D (2003). Effect of hardness on decoction of Chinese medicine. *Journal of Traditional Medicine*.

[13] (1975). *Guide to General Kampo Prescriptions*.

[14] Ikegawa K, Takayanagi H, Anan T (1990). Quantitative analysis of tea constituents, 7: Tannin. *Chagyo Kenkyu Hokoku (Tea Research Journal)*.

[15] Lancheng M, Jingmei L, Duanduan S (2008). The overview of historical evolution and modern research of decoction made from powdered drugs in traditional Chinese medicine. *Chinese Journal of Experimental Traditional Medical Formulae*.

[16] Arichi S, Tani T, Kubo M (1979). Studies on BUPLEURI RADIX and Saikosaponin. (3) Quantitative Analysis of Saikosaponin of Commercial BUPLEURI RADIX. *Medical Journal of Kinki University*.

[17] Zhang X (2011). Decoction method in traditional Chinese medicines. *Chinese Medicine Modern Distance Education of China*.

